# Disruption of *Pseudomonas putida* by high pressure homogenization: a comparison of the predictive capacity of three process models for the efficient release of arginine deiminase

**DOI:** 10.1186/s13568-016-0260-6

**Published:** 2016-10-03

**Authors:** Mahesh D. Patil, Gopal Patel, Balaji Surywanshi, Naeem Shaikh, Prabha Garg, Yusuf Chisti, Uttam Chand Banerjee

**Affiliations:** 1 Department of Pharmaceutical Technology (Biotechnology), National Institute of Pharmaceutical Education and Research, Sector-67, S.A.S. Nagar, Punjab 160062 India; 2 Department of Pharmacoinformatics, National Institute of Pharmaceutical Education and Research, Sector-67, S.A.S. Nagar, Punjab 160062 India; 3 School of Engineering, Massey University, Private Bag 11 222, Palmerston North, New Zealand

**Keywords:** *Pseudomonas putida*, High-pressure homogenization, Arginine deiminase, Response surface method, Artificial neural network, Support vector machine

## Abstract

Disruption of *Pseudomonas putida* KT2440 by high-pressure homogenization in a French press is discussed for the release of arginine deiminase (ADI). The enzyme release response of the disruption process was modelled for the experimental factors of biomass concentration in the broth being disrupted, the homogenization pressure and the number of passes of the cell slurry through the homogenizer. For the same data, the response surface method (RSM), the artificial neural network (ANN) and the support vector machine (SVM) models were compared for their ability to predict the performance parameters of the cell disruption. The ANN model proved to be best for predicting the ADI release. The fractional disruption of the cells was best modelled by the RSM. The fraction of the cells disrupted depended mainly on the operating pressure of the homogenizer. The concentration of the biomass in the slurry was the most influential factor in determining the total protein release. Nearly 27 U/mL of ADI was released within a single pass from slurry with a biomass concentration of 260 g/L at an operating pressure of 510 bar. Using a biomass concentration of 100 g/L, the ADI release by French press was 2.7-fold greater than in a conventional high-speed bead mill. In the French press, the total protein release was 5.8-fold more than in the bead mill. The statistical analysis of the completely unseen data exhibited ANN and SVM modelling as proficient alternatives to RSM for the prediction and generalization of the cell disruption process in French press.

## Introduction

The enzyme arginine deiminase (ADI; E.C. 3.5.3.6) catalyses the irreversible deamination of the guanidine group of l-arginine to citrulline and ammonia (Shirai et al. 2001). ADI is potentially useful in the treatment of certain cancers. Certain tumor cells require an external supply of arginine for rapid proliferation and other functions and ADI-mediated arginine deprivation is effective in the treatment of such tumors (Dillon et al. 2004; Panchaud et al. 2013; Wangpaichitr et al. 2014; Patil et al. 2016a).

ADI-based treatments used in clinical investigations have mostly relied on ADI from *Mycoplasmal* sources (Fayura et al. 2013; Ahn et al. 2014). *Pseudomonas* sp. has been reported as a potential producer of ADI. It was found to be the best organisms for ADI production among the largest number (83 strains of bacteria, 31 strains of yeasts, 15 strains of molds and 15 strains of actinomycetes) of microorganisms screened by Kakimoto et al. (1971). However, most of the studies are confined to *Mycoplasmal* arginine deiminase till date. Very few studies have focused on the bioprocessing aspects of ADI from *Pseudomonas* sp.

As ADI is an intracellular enzyme, its recovery from bacterial cells requires an effective method of cell breakage. High-pressure homogenization of a concentrated bacterial cell suspension in a French-press is an option for cell disruption. In a French-press, the bacterial slurry contained in a steel cylinder is forced through a narrow orifice by means of a piston. The sudden release of pressure as the slurry emerges from the orifice is responsible for cell rupture. No work appears to have been reported on disruption of *Pseudomonas* sp. for release of ADI. The disruption process was investigated in detail in a French-press and compared with the cell disruption performance of a conventional bead mill.

The disruption of the cells was measured in terms of the following responses: (1) release of the intracellular protein; (2) release of ADI; and (3) the fraction of the cells broken. The three main operational factors for a French-press are the concentration of the cells in the suspension being processed, the operational pressure of the press and the number of passes of the cell slurry through the press. The effect of these factors on cell disruption was characterized. A face-centered central composite design (CCD) of experiments with 3-factors measured at 3-levels was used. The data obtained were used to develop response surface models, artificial neural network (ANN) model and support vector machine (SVM) models for predicting the performance of the disruption process.

In conventionally designed experiments, a single factor is varied at a time and the response is measured. This form of experimentation does not allow an estimation of the interactive effect of factors on the measured response. A central composite experiment design in combination with the response surface method (RSM) is more efficient than conventional experiment design and allows interactive effects of factors to be determined (Bandaru et al. 2006; Ghevariya et al. 2011). RSM provides a quantitative relationship (or a model) between the experimental factors and the response. This model equation can be used to predict the response for any combination of the factor values within the experimental space.

A model based on an artificial neural network (ANN) is a potential alternative to an RSM model. The data generated by a set of experiments can be used to train an ANN to predict a response. Such a trained ANN can often effectively predict a future response for any given set of the input variables. This learnt ability to predict does not require any explicit relationships between the inputs and the response (Witek-Krowiak et al. 2014; Maran and Priya 2015); that is, no phenomenological understanding of a process is necessary for ANN-based predictions. ANNs are especially useful for modeling highly nonlinear responses. An alternative to ANN is the use of SVM models to predict a response. SVM algorithms use an experimental data set for supervised learning so that a response can be predicted.

The optimal conditions for the release of the intracellular ADI from *P. putida* KT2440 by disruption in a French-press are reported. The CCD experimental data are used to establish the RSM-based models, the ANN model and the SVM models for predicting the experimental responses (i.e. total protein release, ADI release and the fraction of cells disrupted). The prediction and generalization capabilities of the models are compared.

## Materials and methods

### Chemicals

All chemicals were purchased from Hi-Media Laboratories (Mumbai, India). All reagents and solvents used were of analytical grade.

### Microorganism and cultivation conditions

*Pseudomonas putida* KT2440 was a kind gift from Professor Manfred Zinn, Laboratory for Biomaterials, Empa-Swiss Federal Laboratories for Materials Science and Technology, Switzerland. The stock cultures were maintained on Luria Bertani (LB) medium and stored at −80 °C. The bacterium was grown aseptically in a 14 L stirred-tank bioreactor (BioFlo 310; New Brunswick Scientific, USA) with a working volume of 8 L as previously reported (Patil et al. 2016b).

The culture medium had the following composition (g/L): fructose 30, yeast extract 7.5, bacto peptone 12.5, arginine 4, Na_2_HPO_4_ 3, and NaHPO_4_ 1. The initial pH was adjusted to 7.5. The bioreactor with the culture medium was autoclaved (121 °C, 20 min), cooled to 30 °C and inoculated. The inoculum had been prepared by transferring 100 μL of a stock suspension to 100 mL of LB medium in a 500 mL shake flask and incubating overnight at 30 °C. A 25 mL portion of this overnight culture was transferred to a 1 L shake flask containing 225 mL of the above specified production medium and incubated at 30 °C, 200 rpm, for 12 h. A 800 mL portion of this culture from multiple shake flasks was used to inoculate the bioreactor. The inoculum volume was 10 % of the initial volume of the culture medium.

The bioreactor was agitated at 350 rpm using a Rushton turbine agitator. The pH was measured but not controlled. The temperature was controlled at 30 °C. Filter sterilized air (3.5 L/min, or 0.5 vvm) was continuously sparged through the culture medium. Foaming was controlled by adding sterile polypropylene glycol (Sigma-Aldrich; catalog no. 202339) antifoam agent as needed. The broth was harvested after 28 h of fermentation and the cells were recovered by centrifugation (7000*g*, 20 min, 4 °C). The cell pellet was washed twice with phosphate buffer (50 mM, pH 7.0) and kept refrigerated at 4 °C until needed for disruption.

### Disruption of cells

#### Bead milling

A high speed bead mill (Dyno-mill; Willey A. Bachofen AG Maschinenfabric, Muttenz, Switzerland) was used for cell disruption. The mill consisted of a 300 mL horizontal, cylindrical disruption chamber with a central agitator driven by a variable speed motor. The chamber was filled with glass beads (0.5–0.75 mm in diameter) to 80 % of its nominal volume. Therefore, the bead loading was 80 %.

The cells were suspended in phosphate buffer (50 mM, pH 7.0, containing 0.5 mM phenylmethylsulfonyl fluoride (PMSF) to obtain a final cell concentration of 100 g/L. The cell suspension was cooled to 4 °C, placed in the precooled grinding chamber and ground for 15 min. The agitation speed during grinding was 3000 rpm. The grinding chamber was continuously cooled by circulating cooling water (5–6 °C) through the jacket surrounding the chamber. Samples were taken periodically as grinding progressed.

#### Disruption in French press

A French press (model FA078A with 40 K pressure cell; Thermo Spectronic, Rochester, NY, USA) was used for high-pressure homogenization. For each experiment, 15 mL of cell suspension was prepared at the desired concentration of biomass. PMSF was added to a final concentration of 0.5 mM to suppress protease-mediated degradation of the released proteins. The 40 K pressure cell was precooled to 4 °C before being filled with the bacterial suspension. The cell suspension at 4 °C was then disrupted for a predetermined number of passes. Between passes, the cell slurry was held in an ice bath.

The experimental factors of the number of passes, the processing pressure and the biomass concentration were set at the desired values in keeping with the matrix designed by Design Expert™ 8.0 software (Stat-Ease Inc, USA). The ADI activity, the total protein release and the fraction of the cells disrupted were measured as explained in the following sections.

### Experimental design

#### Response surface method

The response surface method (RSM) was used to examine the dependence of the responses on the process variables. The experimental factors used in the central composite design were: (1) the cell-mass concentration in the slurry being homogenized (*A*, g/L); (2) the disruption pressure setting of the French press (*B*, bar); and (3) the number of passes (*C*). The actual values of factors, the measured responses and the predicted responses are shown in Table 1. The ranges of the factors were selected based on the preliminary studies and the literature values (Singh et al. 2005; Singh 2013). The CCD matrix consisted of 20 experiments (Table 1) (number of experiments = $2^{k} + 2k + \eta_{0}$, where *k* = 3 is the number of factors and *η*_0_ is the number of replicates at the centre point (Tam et al. 2012). The levels of the factors in each experiment were as specified by the Design Expert™ software. The dependent variables, or the responses, were the ADI released, the total protein released and the fraction of the cells disrupted. The response data were fitted to a second order polynomial equation to generate the contour plots.

**Table 1:** Factor values and responses in the various runs

Run	*A* (g/L)	*B* (bar)	*C*	ADI activity (U/mL)				Total protein release (g/L)				Cell disruption (%)			
				Actual	RSM predicted	ANN predicted	SVM predicted	Actual	RSM predicted	ANN predicted	SVM predicted	Actual	RSM predicted	ANN predicted	SVM predicted
1	200	413.7	2	22.5	23.2	23.0	22.6	2.7	2.6	3.0	2.8	63.3	63.9	64.6	63.4
2	100	965.3	1	20.1	20.1	17.8	20.2	2.8	2.8	2.9	2.9	75.5	73.6	76.3	75.4
3	300	413.7	3	22.6	22.5	23.6	22.5	4.9	4.8	4.3	4.8	70.2	71.9	77.7	70.3
4	300	965.3	1	23.2	23.1	23.7	23.3	4.7	4.6	4.5	4.6	65.5	66.4	67.3	65.4
5	100	965.3	3	22.5	22.8	21.5	22.6	2.4	2.3	2.8	2.4	93.0	94.5	89.3	92.9
6	100	689.5	2	19.5	19.4	17.2	19.6	2.2	2.2	2.8	2.1	86.4	87.3	80.7	86.5
7	300	689.5	2	24.4	24.3	23.6	24.8	4.0	4.2	4.4	4.2	78.9	79.0	73.2	78.9
8	200	689.5	1	25.5	26.3	24.9	25.6	3.1	3.0	2.9	2.9	57.8	60.0	58.3	57.9
9	200	689.5	2	25.2	25.0	25.2	25.1	3.5	3.0	3.1	3.0	80.7	79.7	79.5	79.7
10	300	689.5	3	22.1	22.6	22.6	22.2	5.5	5.4	4.7	5.4	85.4	84.5	83.9	85.3
11	200	689.5	2	25.4	25.0	25.2	25.1	3.1	3.0	3.1	3.0	84.3	79.7	79.5	79.7
12	200	689.5	2	25.0	25.0	25.2	25.1	2.8	3.0	3.1	3.0	79.6	79.7	79.5	79.7
13	300	413.7	1	27.7	27.4	26.1	27.6	3.1	3.2	3.8	3.2	43.1	41.3	42.4	43.0
14	200	689.5	3	26.1	25.2	25.0	24.7	3.3	3.6	3.4	3.5	86.7	85.7	86.7	86.8
15	200	965.3	2	25.3	24.5	24.8	25.2	3.1	3.3	3.6	3.4	82.5	83.1	84.9	82.6
16	200	689.5	2	23.7	25.0	25.2	25.1	2.8	3.0	3.1	3.0	76.7	79.7	79.5	79.7
17	100	413.7	1	18.0	17.5	19.0	17.9	2.0	2.1	2.3	2.1	47.1	47.8	48.3	47.2
18	200	689.5	2	25.0	25.0	25.2	25.1	3.0	3.0	3.1	3.0	80.0	79.7	79.5	79.7
19	200	689.5	2	25.1	25.0	25.2	25.1	3.1	3.0	3.1	3.0	79.0	79.7	79.5	79.7
20	100	413.7	3	15.7	15.8	17.0	15.8	2.4	2.4	2.7	2.4	82.3	81.2	84.0	82.2

*A*, Cell mass concentration; *B*, Operating pressure; *C*, number of passes

#### Artificial neural network

A trained ANN model predicts responses (outputs) based on the input values of the factors without defining an explicit relationship between inputs and outputs. ANN is universally applicable for modeling nonlinear responses whereas RSM approximates responses in terms of an explicit quadratic function of the independent experimental factors (Marchitan et al. 2010; Maran and Priya 2015). The ANN used here was a three-layered feed-forward neural network consisting of an input layer, a hidden middle layer and an output layer. Neural Network Toolbox of MATLAB (version 7.8.0; http://www.mathworks.com) was used in development of the ANN model. Tangent sigmoid transfer functions (tansig) were used at the hidden layer and linear transfer functions (purelin) were used at output layer. A back propagation training process was used in which the weights of the connections between the nodes of the layers were repetitively adjusted so that the output value was as close as possible to the desired output. The neural network was fed with values of the three input variables (i.e. cell-mass concentration, the operating pressure and the number of passes). Therefore, the number of neuron in the input layer was three. Each input value was normalized by dividing by the maximum value for the specific input. The output layer had three neurons corresponding to the ADI released, the total protein released and the fraction of cells disrupted.

#### Support vector machine (SVM)

SVM is a supervised learning algorithm with an excellent capability for global optimization. In contrast with ANN models, a SVM solution is global and unique (Smola and Scholkopf 2004). As an important advantage, SVM can be implemented using a small number of samples for reliable prediction of the responses (Caydas and Ekici 2012). In the present study, experimental data (Table 1) was used to develop the SVM model with a radial basis function.

#### Comparison of the models

Evaluation of the goodness of fit and prediction capacity of the constructed models was performed by error analyses. Root mean square error (RMSE), standard error of prediction (SEP), relative percent deviation (RPD) and determination coefficient (*R*^2^) values were used to compare the measured and the predicted responses. The following equations were used in the calculations (Bingöl et al. 2012; Geyikçi et al. 2012):

1 $$R^{2} \; = \;1\; - \;\frac{{\mathop \sum \nolimits_{i = 1}^{n} \;\left( {Y_{ip} \; - \;Y_{ie} } \right)^{2} }}{{\mathop \sum \nolimits_{i = 1}^{n} \;\left( {Y_{ie} \; - \;Y_{e} } \right)^{2} }}$$

2 $${\text{RMSE}}\; = \;\sqrt {\frac{{\mathop \sum \nolimits_{i = 1}^{n} \;\left( {Y_{ie} \; - \;Y_{ip} } \right)^{2} }}{n}}$$

3 $${\text{SEP}}\; = \;100\; \times \;\frac{\text{RMSE}}{{Y_{e} }}$$

4 $${\text{RPD}}\; = \;\frac{100}{n}\;\mathop \sum \limits_{i = 1}^{n} \;\frac{{\left| {\left( {Y_{ip} \; - \;Y_{ie} } \right)} \right|}}{{\left| {Y_{ie} } \right|}}.$$

In the above equations, *Y*_*ie*_ is the experimental data, *Y*_*ip*_ is the corresponding predicted data, *Y*_*e*_ is the mean value of experimental dataset and *n* is the number of measurements in the experimental dataset. In general, small values of RMSE and SEP suggest a good ability of a model to predict the experimental data.

### Analytical methods

#### ADI activity

The ADI activity was determined using a modification of an earlier published method (Ni et al. 2011). Briefly, 100 µL of the supernatant of the centrifuged cell homogenate was incubated (37 °C, 30 min) with 30 mM of l-arginine and 200 mM sodium phosphate buffer (pH 6.0) in a final volume of 1 mL. This reaction mixture was incubated at 37 °C for 30 min. A 100 µL of the reaction mixture was used to measure the ADI activity. One unit of ADI activity was defined as the amount of enzyme that converted 1 µmol of l-arginine to 1 µmol of l-citrulline per min at the incubation temperature.

The citrulline produced was quantified using the diacetyl monoxime thiosemicarbazide (DAM) method (Boyde and Rahmatullah 1980). For this, a calibration curve was produced by diluting a 3 mM standard solution of l-citrulline to various concentrations (0.25–3.00 mM). A 100 µL portion of this diluted solution was mixed with 3 mL of a chromogenic reagent. The mixture was incubated at 95 °C for 5 min, cooled, and the spectrophotometric absorbance was measured at 530 nm against a blank of distilled water treated the same way as the l-citrulline standard solution. The measured absorbance was plotted against the known concentration of l-citrulline to obtain a calibration curve.

The chromogenic reagent was prepared just before use by adding 5 mg of thiosemicarbazide to 50 mL of diacetyl monoxime solution (500 mg of diacetyl monoxime dissolved in 100 mL of distilled water) and mixing in 100 mL of acid-ferric solution. The latter had been made by adding 25 mL of concentrated sulfuric acid and 20 mL of concentrated phosphoric acid to 55 mL of distilled water, cooling to room temperature, and dissolving 25 mg of ferric chloride (Patil et al. 2016b). Assays were carried out in duplicate and the mean values were used for building ANN and SVM models.

#### Protein quantification

The total protein released was quantified using the Bradford method (Bradford 1976) with bovine serum albumin as the standard. Optical density was read at 595 nm. Measurements were in triplicate and mean values are reported.

#### The extent of cell disruption

The extent of cell disruption was quantified as the fraction of the cells disrupted (*F*_*d*_) (Tam et al. 2012), calculated as follows:

5 $$F_{d} \;\left( \% \right)\; = \;\left( {1\; - \;\frac{{I_{a} }}{{I_{b} }}} \right)\; \times \;100.$$

In the above equation *I*_*b*_ is the intact cell concentration before disruption and *I*_*a*_ is the intact cell concentration after the disruption treatment. Values of *I*_*b*_ and *I*_*a*_ were determined by measuring the optical density at 600 nm (OD_600_) before and after passage of a sample through the French press.

## Results

### Optimization using RSM

The actual values of the three independent variables were optimized using a central composite design. The optimization aimed to separately maximize the responses of the extent of cell disruption, the total protein release and ADI release. A total of 20 runs were carried out with the actual values of the factors, the actual responses and the predicted responses as in Table 1. The predicted and the measured responses were in good agreement.

The experimental responses and the coded values of the factors fitted the following second order polynomials:

6 $$Y_{1} \; = \;24.96\; + \;2.43A\; + \;0.67B\; - \;0.55C\; - \;1.72AB\; - \;0.80AC\; + \;1.10BC\; - \;3.10A^{2} \; - \;1.10B^{2} \; + \;0.75C^{2}$$

7 $$Y_{2} \; = \;2.99\; + \;1.04A\; + \;0.33B\; + \;0.29C\; + \;0.17AB\; + \;0.32AC\; - \;0.21BC\; + \;0.21A^{2} \; - \;0.035B^{2} \; + \;0.28C^{2}$$

8 $$Y_{3} \; = \;79.65\; - \;4.12A\; + \;9.59B\; + \;12.86C\; - \;0.17AB\; - \;0.71AC\; - \;3.13BC\; + \;3.51A^{2} \; - \;6.20B^{2} \; - \;6.84C^{2}$$

In the above equations *Y*_1_ is the ADI released (U/mL); *Y*_2_ is the total protein released (g/L); *Y*_3_ is the fraction of the cells disrupted (%); *A* is the cell-mass concentration (g/L); *B* is the disruption pressure (bar); and *C* is the number of passes.

The adequacy of fit of the above models (Eqs. 6–8) was tested by analysis of variance (ANOVA) (Tables 2, 3, 4). The above models were found to be adequate for the specified responses. The ANOVA for the model for ADI release (Eq. 6) is shown in Table 2. The correlation coefficient (*R*) of the model was 0.985, indicating a good agreement between the experimental data and the model-predicted values. The determination coefficient (*R*^*2*^) value was 0.9698; indicating that nearly 97 % of the variation in ADI release could be attributed to the experimental factors and only 3 % of the total variation in the measured data could not be explained by the model.

**Table 2:** Analysis of variance for the response surface quadratic model (Eq. 6) for ADI release

Source	SS	DF	MS	*F* value	*P* value (Prob > F)
Model	163.87	9	18.21	35.67	<0.0001
Cell concentration (*A*)	59.10	1	59.10	115.79	<0.0001
Operating pressure (*B*)	4.52	1	4.52	8.86	0.0139
Number of passes (*C*)	3.05	1	3.05	5.98	0.0345
*AB*	23.79	1	23.79	46.61	<0.0001
*AC*	5.15	1	5.15	10.09	0.0099
*BC*	9.72	1	9.72	19.04	0.0014
*A* ^2^	26.36	1	26.36	51.64	<0.0001
*B* ^2^	3.36	1	3.36	6.58	0.0281
*C* ^2^	1.53	1	1.53	2.99	0.1142
Residual	5.10	10	0.51		
Lack-of-fit	3.33	5	0.67	1.88	0.2529
Pure error	1.77	5	0.35		
Cor. Total	168.98	19			

*R* ^2^ = 0.9698; adjusted *R* ^2^ = 0.9426; predicted *R* ^2^ = 0.8297; adequate precision = 23.003; coefficient of variation (%) = 3.08; standard deviation = 0.71; mean = 23.23

*SS* sum of squares; *DF* degrees of freedom; *MS* mean sum of squares

**Table 3:** Analysis of variance for the response surface quadratic model (Eq. 7) of total protein release

Source	SS	DF	MS	*F* value	*P* value (Prob > F)
Model	14.95	9	1.66	29.01	<0.0001
Cell concentration (*A*)	10.82	1	10.82	189.04	<0.0001
Operating pressure (*B*)	1.07	1	1.07	18.71	0.0015
No. of passes (*C*)	0.81	1	0.81	14.19	0.0037
*AB*	0.22	1	0.22	3.81	0.0796
*AC*	0.80	1	0.80	13.93	0.0039
*BC*	0.34	1	0.34	5.88	0.0358
*A* ^2^	0.12	1	0.12	2.14	0.1740
*B* ^2^	3.321 × 10^−3^	1	3.321 × 10^−3^	0.058	0.8145
*C* ^2^	0.22	1	0.22	3.88	0.0772
Residual	0.57	10	0.057		
Lack-of-fit	0.27	5	0.054	0.88	0.5535
Pure error	0.30	5	0.061		
Cor. total	15.52	19			

*R* ^2^ = 0.9631; adjusted *R* ^2^ = 0.9299; predicted *R* ^2^ = 0.8457; adequate precision = 19.536; coefficient of variation (%) = 7.42; standard deviation = 0.24; mean = 3.22

*SS* sum of squares; *DF* degrees of freedom; *MS* mean sum of squares

**Table 4:** Analysis of variance for the response surface quadratic model (Eq. 8) of the extent of cell disruption

Source	SS	DF	MS	*F* value	*P* value (Prob > F)
Model	3292.93	9	365.88	67.15	<0.0001
Cell concentration (*A*)	170.08	1	170.08	31.22	0.0002
Operating pressure (*B*)	920.30	1	920.30	168.90	<0.0001
Number of passes (*C*)	1653.15	1	1653.15	303.41	<0.0001
*AB*	0.24	1	0.24	0.044	0.8378
*AC*	4.04	1	4.04	0.74	0.4091
*BC*	78.15	1	78.15	14.34	0.0036
*A* ^2^	33.88	1	33.88	6.22	0.0318
*B* ^2^	105.55	1	105.55	19.37	0.0013
*C* ^2^	128.56	1	128.56	23.60	0.0007
Residual	54.49	10	5.45		
Lack-of-fit	23.64	5	4.73	0.77	0.6112
Pure error	30.84	5	6.17		
Cor. total	3347.42	19			

*R* ^2^ = 0.9837; adjusted *R* ^2^ = 0.9691; predicted *R* ^2^ = 0.8711; adequate precision = 32.201; coefficient of variation (%) = 3.12; standard deviation = 2.33; mean = 74.89

*SS* sum of squares; *DF* degrees of freedom; *MS* mean sum of squares

A high value of the adjusted determination coefficient (=0.9426) in the present model (Eq. 6) confirmed the model to be significant. The Fisher’s *F* test (*F* values), calculated as the ratio between the lack-of-fit mean square and the pure error mean square, was used to verify the competence of the factors in describing the variation in the data about its mean value. The *F* value for the present model (i.e. Eq. 6) was 35.67 (Table 2), suggesting that the model was significant and the probability of the *F* value of the model being due to experimental noise was only 0.01 %.

The ‘adequate precision value’ is an index of the signal-to-noise ratio and its value must be greater than 4 for a model to be considered a good fit to the data. Adequate precision value of Eq. (6) was 23.003 (Table 2), suggesting the model to be satisfactory for navigating the design space. A low pure error value (=1.77, Table 2) suggested a good reproducibility of the experimental responses.

Similarly, the models for total protein release (Eq. 7) and the fraction of cells disrupted (Eq. 8) were adequate as revealed by ANOVA (Tables 3, 4). The correlation coefficient values for the models were high (>0.99). A good agreement of the model predictions and the measured data is shown for the two models (Eq. 7, 8).

**Fig. 1:**
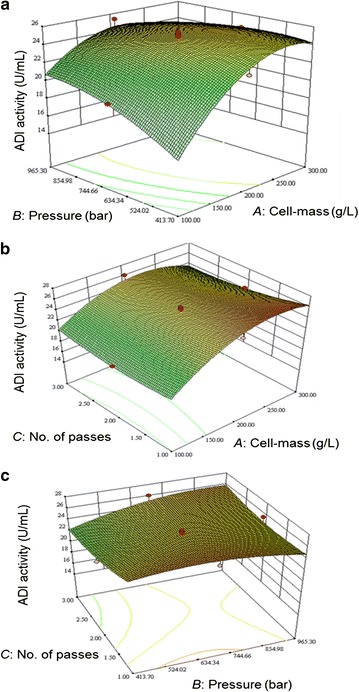
Response surface plots showing the interactive effect of the following factors on ADI release: **a** cell-mass concentration and the operating pressure (the number of passes was fixed at 2); **b** cell-mass concentration and the number of passes (the operating pressure was fixed at 689.5 bar); and **c** operating pressure and the number of passes (the cell mass concentration was fixed at 200 g/L)

**Fig. 2:**
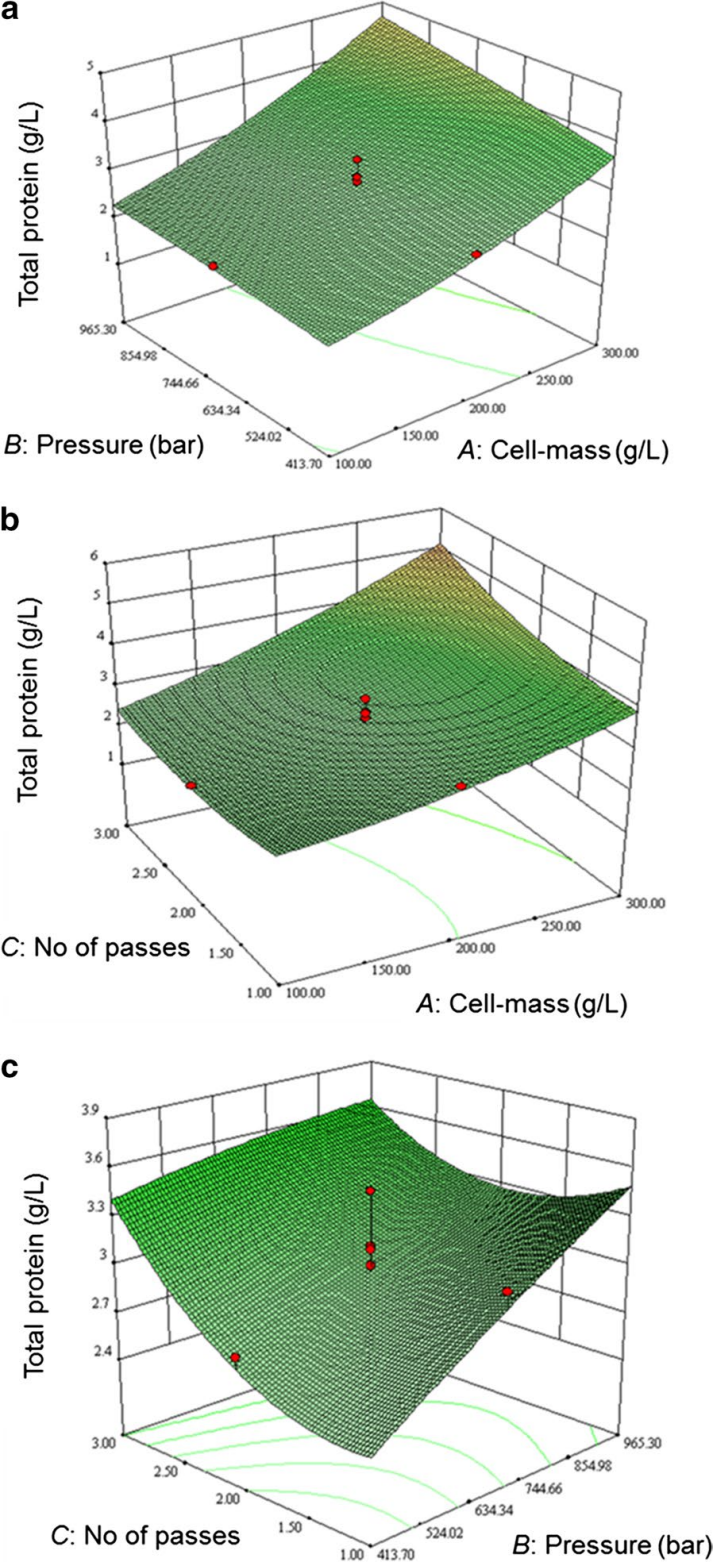
Response surface plots showing the interactive effect of the following factors on total protein release: **a** cell-mass concentration and the operating pressure (the number of passes was fixed at 2); **b** cell-mass concentration and the number of passes (the operating pressure was fixed at 689.5 bar); and **c** operating pressure and the number of passes (the cell mass concentration was fixed at 200 g/L)

**Fig. 3:**
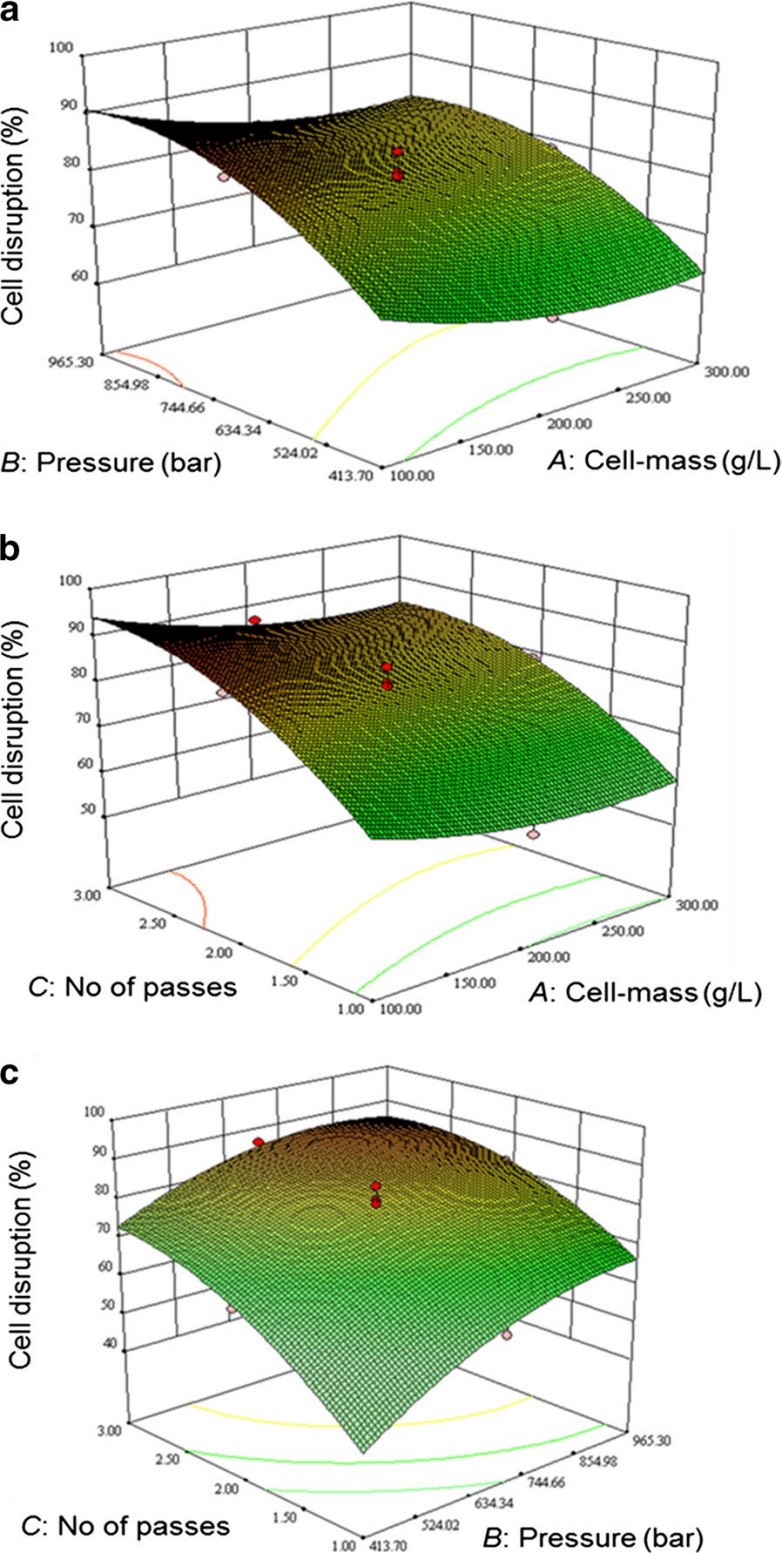
Response surface plots showing the interactive effect of the following factors on the fraction of cells disrupted: **a** cell-mass concentration and the operating pressure (the number of passes was fixed at 2); **b** cell-mass concentration and the number of passes (the operating pressure was fixed at 689.5 bar); and **c** operating pressure and the number of passes (the cell mass concentration was fixed at 200 g/L)

For ADI release response (Eq. 6; Table 2), all the linear terms (*A*, *B* and *C*) and interactive terms (*AB*, *AC* and *BC*) were significant. Only one quadratic term (i.e. *C*^2^, Table 2) was not significant. For the total protein release response (Eq. 7), none of the quadratic terms was significant (Table 3) and the interactive term *AB* was also not significant (Table 3). For the fraction of cells disrupted response (Eq. 8), all the linear and quadratic terms were significant (Table 4), but two of the interactive terms (i.e. *AB* and *AC*) were not significant. Thus, all the experimental factors individually had a significant effect on the cell fraction disrupted, the total protein release and the release of ADI. Cell mass concentration in the slurry had the strongest effect on the total protein release. The interactive effects of the factors on the various responses are shown in Figs. 1, 2 and 3.

### Artificial neural network modelling

**Fig. 4:**
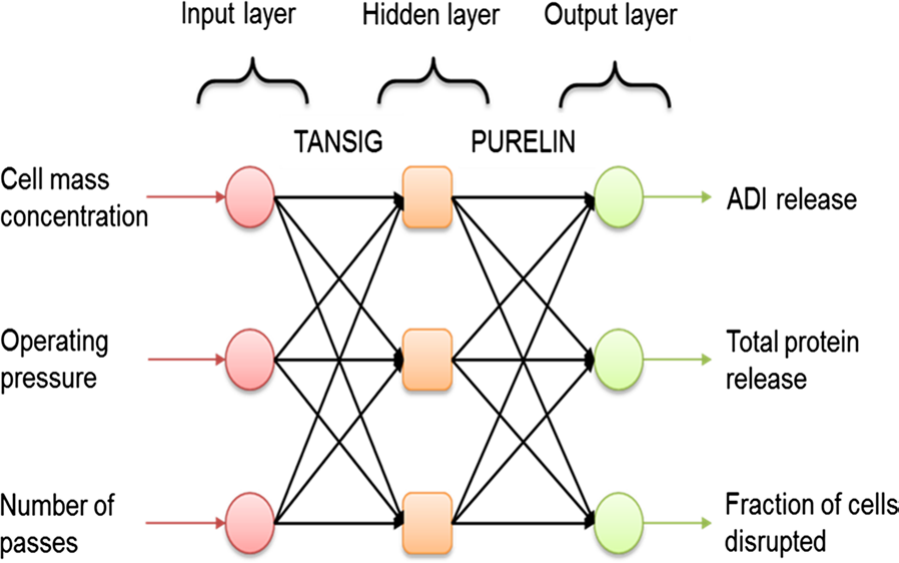
Architecture of ANN model used

A feed forward back propagation ANN algorithm was used as it has been found to be broadly useful for modeling bioprocesses (Sarve et al. 2015). Levenberg–Marquardtback-propagation algorithm (trainlm) was used as the training function in view of its known good performance (Maran and Priya 2015; Sarve et al. 2015). The optimum number of neurons and transfer functions for the hidden layer was determined based on minimizing the value of the mean squared error (MSE) of the training and prediction datasets. As the training dataset was small, the number of neurons in the hidden layer was optimized only in the range of one and three. The MSE was found to be the minimum for three neurons and tansig transfer function in the hidden layer. Hence, a feed-forward neural network with three neurons each in the input layer, the output layer and the hidden layer was used for modeling. Purelin transfer function at the output layer was used. Figure 4 shows the optimum model architecture.

**Fig. 5:**
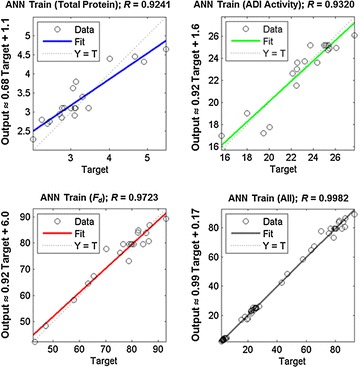
Comparison between ANN-predicted responses and the measured responses

For evaluating the model, the experimental values of the responses were compared with the corresponding predicted values. The relevant data are shown in Table 1 for the responses of ADI release, the total protein release and the fraction of the cells disrupted. As shown in Fig. 5, the ANN model with the training dataset had very good *R* values of 0.932, 0.924 and 0.972, respectively, for ADI released, total protein released and *F*_*d*_. The *R* value for the entire dataset (i.e. the three datasets combined) was also good (=0.998). Therefore, the ANN model trained using the experimental data, was precise enough for predicting the disruption responses.

### Support vector machine model

The experimental data (Table 1) was used to develop the SVM models. A radial basis function (RBF) was used as kernel for model development because it is effective and speeds up the training process. The input variables were the normalized values of the cell-mass concentration, the operating pressure and the number of passes. Each input was normalized by dividing with the largest value in the input series. As a SVM operates only on single output, three different models were developed for the outputs of ADI release, the total protein release and the fraction of the cells disrupted.

**Fig. 6:**
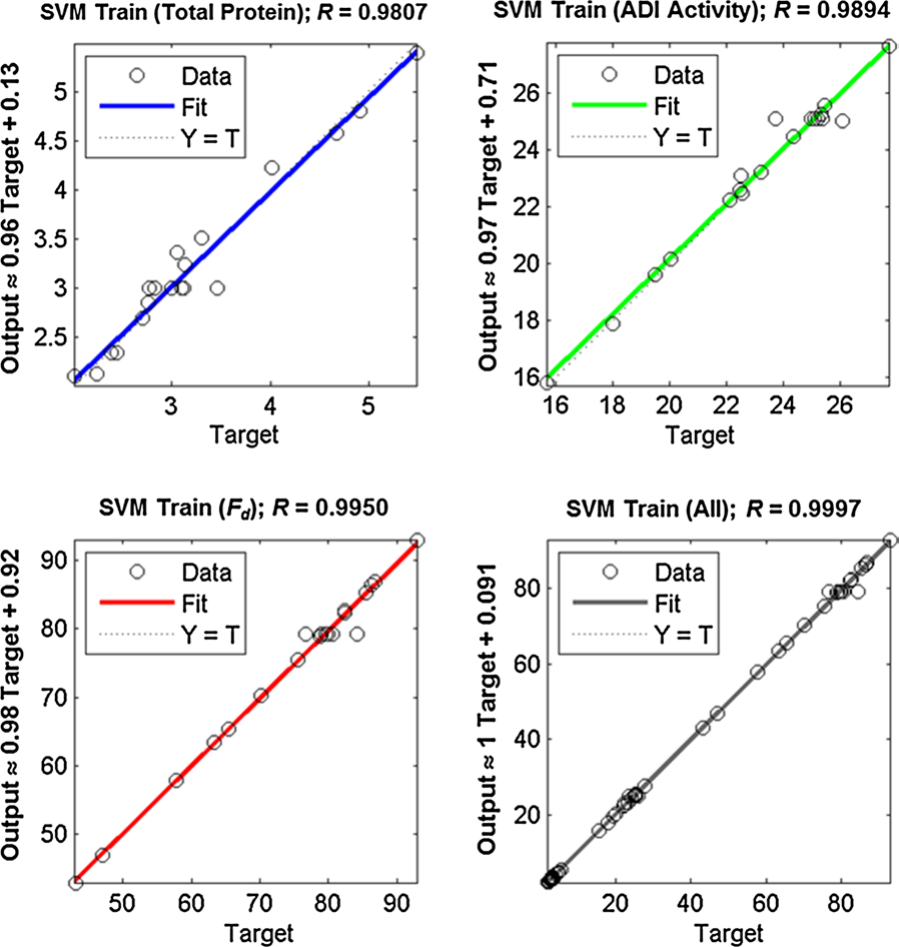
Comparison between SVM-predicted response and the measured responses

In developing a SVM model with a RBF kernel, the penalty parameter α and the kernel coefficient γ need to be optimized. Values of α and γ for each model were determined by a systematic grid search. The software scikit-learn 0.17 (http://scikit-learn.org) (Pedregosa et al. 2011) was used for development of the models. As shown in Fig. 6, the fit of the experimental data with the SVM-predicted responses was good: the *R* values of the fit were 0.989, 0.981, 0.995 and 0.999 for the ADI release, the total protein release, the fraction of cells disrupted and the training datasets, respectively.

### Comparison of predictive and generalization capacities of the models

The experimental responses and the predictions of the various models for the unseen datasets are shown in Table 5. The unseen dataset consisted of the results of 8 new experiments that were not used in any model development.

**Table 5:** Experimental and predicted responses for the unseen dataset

Run	*A* (g/L)	*B* (bar)	*C*	ADI activity (U/mL)				Total protein release (g/L)				Cell disruption (%)			
				Actual	RSM predicted	ANN predicted	SVM predicted	Actual	RSM predicted	ANN predicted	SVM predicted	Actual	RSM predicted	ANN predicted	SVM predicted
1	260	510.2	1	27.2	27.6	26.4	27.3	3.1	3.1	3.4	3.1	46.1	49.9	47.1	47.3
2	235	689.5	1	26.4	27.0	26.2	26.5	3.3	3.3	3.3	3.2	56. 4	59.2	56.3	57.2
3	210	792.9	3	24.8	25.7	24.4	25.4	3.7	3.8	3.8	3.7	80.2	86.7	86.2	87.9
4	250	489.5	1	26.7	27.5	26.4	27.1	3.5	3.0	3.2	3.0	43.1	46.7	47.1	45.6
5	260	413.7	1	26.6	27.5	26.5	27.1	3.3	2.9	3.2	2.9	36.0	40.4	44.6	40.8
6	240	861.8	2	24.0	25.0	24.1	25.6	3.7	3.7	4.0	3.7	81.0	82.1	81.3	82.1
7	240	827.4	1	24.7	26.2	25.5	25.8	3.8	3.6	3.7	3.6	62.6	64.5	63.5	62.5
8	200	861.8	3	25.01	25.8	24.5	25.5	4.1	3.6	3.7	3.6	81.2	87.3	86.7	88.2

The predictive performances of the developed models were compared on the basis of RMSE, the standard error of prediction (SEP), the MSE, and the relative percent deviation (RPD). These statistical parameters are reported in Table 6 both for the training datasets and the unseen datasets.

**Table 6:** Validation and comparison with the unseen dataset

Statistical parameter	ADI activity (U/mL)			Total protein release (g/L)			Cell disruption (%)		
	RSM	ANN	SVM	RSM	ANN	SVM	RSM	ANN	SVM
*Training dataset*									
*R* ^2^	0.970	0.869	0.975	0.963	0.854	0.960	0.984	0.945	0.991
MSE	0.255	1.175	0.208	0.029	0.151	0.031	2.725	9.298	1.586
RMSE	0.505	1.084	0.456	0.170	0.389	0.177	1.651	3.049	1.259
SEP	2.175	4.665	1.964	5.264	12.073	5.496	2.204	4.071	1.682
RPD	1.573	3.978	1.046	4.272	10.211	4.463	1.790	2.910	0.694
*Unseen dataset*									
*R* ^2^	0.943	0.845	0.892	0.584	0.533	0.640	0.990	0.969	0.979
MSE	0.866	0.218	0.608	0.083	0.049	0.081	17.469	19.948	17.720
RMSE	0.931	0.467	0.780	0.288	0.222	0.285	4.180	4.466	4.209
SEP	3.628	1.821	3.039	8.116	6.264	8.053	6.873	7.344	6.922
RPD	3.469	1.523	2.509	5.966	5.154	5.703	6.728	6.478	5.384

*R* ^2^ determination coefficient; *MSE* mean square error; *RMSE* root mean square error; *SEP* standard error of prediction; *RPD* relative percent deviation; *A* Cell mass concentration; *B* operating pressure; *C* number of passes

Looking only at the training datasets (Table 6, upper panel), the SVM model was superior to the other models for the ADI activity response because it had the lowest values of RMSE, SEP and RPD. Similarly, SVM model was better than the other models for the fraction of cell disrupted response. However, for the total protein release response, the RSM model was better than the other models (Table 6, upper panel).

Based on the unseen data for ADI activity, the MSE, RMSE, SEP and RPD values for the ANN model were lower than for the RSM and SVM predictions (Table 6, lower panel). Therefore, the ANN model was superior to the other models. Similarly, for the total protein release, the ANN model was superior to the other models although the *R*^2^ values of all models were low (Table 6, lower panel). For fraction of the cells disrupted, the SVM model may be considered better than the other models in view of its lower RPD (Table 6, lower panel). (The RMSE and SEP of the SVM model were about the same as the next best model, the RSM).

## Discussion

Although like many bacteria (Hughes et al. 1971; Chisti and Moo-Young 1986; Benov and Al-Ibraheem 2002; Donsì et al. 2009), *P. putida* cells are routinely disrupted in the laboratory using French-press, no detailed analysis of this disruption method has been published.

In the present study, cell mass concentration in the slurry had the strongest effect on the total protein release (Fig. 2a, b) and the ADI release (Fig. 1a, b) mainly because more of these components were available to be released in more concentrated slurry of cells. The biomass specific protein release did not increase with increasing concentration of the cells in the slurry; therefore disruption was actually less effective at higher biomass concentrations. In other types of high-pressure homogenizers, the disruption rate of cells has been generally found to be independent of the cell concentration in the slurry (Singh et al. 2005; Tam et al. 2012; Singh 2013), but the amount of the intracellular material released is obviously dependent on the concentration of the cells in the slurry. In contrast to high-pressure homogenizers, the disruption rate in bead mills is generally observed to increase with increasing concentration of the cells in the slurry being processed (Chisti and Moo-Young 1986; Ricci-Silva et al. 2000; Mei et al. 2005).

For otherwise fixed conditions, the fraction of cells disrupted increased with increasing number of passes but each subsequent pass was less effective in disrupting the cells. This suggests that the extent of disruption in a given pass depended on the concentration of undisrupted cells. Release of intracellular proteins and DNA may also have contributed to progressively reducing the disruption efficacy of a given pass. This is because release of the intracellular polymers increases the viscosity of the cell homogenate (Chisti and Moo-Young 1986) and this reduces flow rate through the homogenizing orifice.

Among the three factors tested, the operating pressure was highly influential in affecting the fraction of the cells disrupted. This was consistent with similar findings for other kinds of high-pressure cell homogenizers. For example, a 4-fold increase in the degree of cell disruption was found in the homogenization of baker’s yeast when the operating pressure was increased from 500 to 2500 bar (Donsì et al. 2009). In the present study, nearly 93 % of the cells in a slurry with a biomass concentration of 100 g/L could be disrupted in three passes at an operating pressure of ~965 bar (run 3, Table 1). This is consistent with similar findings for other Gram negative bacteria. For example, in a French press operated at 689.5 bar more than 98 % release of the intracellular aspartase from *Escherichia coli* K-12 was reported within a single pass using cell slurries with biomass concentrations in the range of 50–250 g/L (Singh 2013).

*Pseudomonas putida* KT2440 is a Gram negative motile bacterium. The cell is approximately 1 μm wide and 2 µm long (Auerbach et al. 2000). Gram negative bacteria generally have a much thinner cell wall than gram positive bacteria and, therefore, are easier to break. Therefore, a combination of higher operating pressures and the number of passes may be necessary for effective use of the French press with other microorganisms. For example, microalgae such as *Chlorella vulgaris* and *Monodus subterraneus* can be far more difficult to break than bacteria such as *P. putida*. Similarly, yeasts such as *S. cerevisae* can be difficult to break as they have much thicker walls (100–200 nm thick) than gram negative bacteria. Notwithstanding its relative robustness, nearly 70 % disruption of a *S. cerevisae* slurry with 600–700 g biomass (fresh weight) per L has been reported within a single pass in a French press but at a much higher operating pressure of 1379 bar (Hughes et al. 1971) than used in the present work. In another study of disruption of a yeast (*Candida* sp.), French press was found to be quite effective in releasing intracellular protein, but required a high operating pressure of 1241 bar (Okungbowa et al. 2007).

Intuitively, a relatively large cell may be expected to be more easily broken than smaller cells, but in practice this is not so. For example, the diameter of *S. cerevisae* cells is much larger (=3.5–5.6 μm depending on age) (Johnston et al. 1979) than of *P. putida* or similarly sized Gram negative bacterium *E. coli* (0.5 μm in width, 2 μm in length), but *S. cerevisiae* is much harder to disrupt as shown by the data above.

Using cell slurry with a biomass concentration of 100 g/L, as was used also with the bead mill in the current work, the ADI release in the French press was 22.5 U/mL (run 5, Table 1). This was 2.7-fold higher than was achieved with the bead mill. The ADI and total protein release after 15 min of bead milling was found to be 8.46 U/mL and 0.48 g/L, respectively (unpublished data). Similarly, with the 100 g/L cell slurry, the maximum protein release with the French press was 2.8 g/L (run 2, Table 1). This value was 5.8-fold greater than in the bead mill. Therefore, in terms of the key parameters of ADI release and protein release, the French press was substantially better than the bead mill. This suggests that any large scale disruption process for *P. putida* should consider using high-pressure homogenization instead of bead milling.

A French press is a batch device with a low throughput. It is suitable only for a sample size of ≤250 mL and cannot be scaled up directly. Nonetheless, it allows for estimating the best operating conditions to use for initial disruption testing in a large-scale high-pressure homogenizer of a similar operating principle as the French press. Such industrial scale homogenizers are available and can be operated in a continuous flow mode with a high throughput (Chisti and Moo-Young 1986; Middelberg 2000).

While high-pressure homogenization is generally suitable for recovering intracellular enzymes, it has the potential to damage certain enzymes by exposing them to intense mechanical shear forces (Chisti and Moo-Young 1986). Indeed, some enzymes of *Pseudomonas aeruginosa* have been shown to be susceptible to such damage, therefore there is the potential for similar damage in enzyme recovery from *P. putida* although for ADI, no such damage was observed.

A near complete disruption of *P. putida* KT2440 cells was achieved in the French press within 3-pases at an operating pressure of ~965 bar for a slurry with a biomass concentration of 100 g/L. At the same operating pressure, essentially complete release of the ADI activity could be achieved in a single pass. Of the models evaluated, ANN model was best for predicting the release of ADI and total protein for a given set of operating variables. The RSM model was best for predicting the fraction of the cells disrupted. The SVM was comparable to RSM for predicting the fraction of the cells disrupted. The conditions that maximized the ADI release were the following: an initial cell concentration of 260 g/L in the slurry; an operating pressure of 510 bar; and a single pass through the machine. With these conditions the ADI release exceeded 27 U/mL and the total protein release exceeded 3 g/L. French press proved to be substantially better than the bead mill in releasing both ADI and total protein from the cells.

## Abbreviations

ADI: arginine deiminase
RSM: response surface methodology
CCD: centre composite design
ANN: artificial neural network
SVM: support vector machine
PMSF: phenylmethylsulfonyl fluoride
MSE: mean square error
RMSE: root mean square error
SEP: standard error of prediction
RPD: relative percent deviation

## References

[CR1] Ahn K-Y, Lee B, Han K-Y, Song J-A, Lee J (2014). Synthesis of *Mycoplasma* arginine deiminase in *E. coli* using stress-responsive proteins. Enzyme Microb Technol.

[CR2] Auerbach ID, Sorensen C, Hansma HG, Holden PA (2000). Physical morphology and surface properties of unsaturated *Pseudomonas putida* biofilms. J Bacteriol.

[CR3] Bandaru VVR, Somalanka SR, Menduc DR, Madicherla NR, Chityala A (2006). Optimization of fermentation conditions for the production of ethanol from sago starch by co-immobilized-amyloglucosidase and cells of *Zymomonas mobilis* using response surface methodology. Enzyme Microb Technol.

[CR4] Benov L, Al-Ibraheem J (2002). Disrupting *Escherichia coli*: a comparison of methods. J Biochem Mol Biol.

[CR5] Bingöl D, Hercan M, Elevli S, Kılıç E (2012). Comparison of the results of response surface methodology and artificial neural network for the biosorption of lead using black cumin. Bioresour Technol.

[CR6] Boyde TRC, Rahmatullah M (1980). Optimization of conditions for the calorimetric determination of citrulline using diacetyl monoxime. Anal Biochem.

[CR7] Bradford MM (1976). A rapid and sensitive method for the quantitation of microgram quantities of protein utilizing the principle of protein-dye binding. Anal Biochem.

[CR8] Caydas U, Ekici S (2012). Support vector machines models for surface roughness prediction in CNC turning of AISI 304 austenitic stainless steel. J Intell Manuf.

[CR9] Chisti Y, Moo-Young M (1986). Disruption of microbial cells for intracellular products. Enzyme Microb Technol.

[CR10] Dillon BJ, Prieto VG, Curley SA, Ensor CM, Holtsberg FW, Bomalaski JS, Clark MA (2004). Incidence and distribution of argininosuccinate synthetase deficiency in human cancers: a method for identifying cancers sensitive to arginine deprivation. Cancer.

[CR11] Donsì F, Ferrari G, Lenza E, Maresca P (2009). Main factors regulating microbial inactivation by high-pressure homogenization: operating parameters and scale of operation. Chem Eng Sci.

[CR12] Fayura LR, Boretsk YR, Pynyaha YV, Wheatley DN, Sibirny AA (2013). Improved method for expression and isolation of the *Mycoplasma hominis* arginine deiminase from the recombinant strain of *Escherichia coli*. J Biotechnol.

[CR13] Geyikçi F, Kılıç E, Çoruh S, Elevli S (2012). Modelling of lead adsorption from industrial sludge leachate on red mud by using RSM and ANN. Chem Eng J.

[CR14] Ghevariya CM, Bhatt JK, Dave BP (2011). Enhanced chrysene degradation by halotolerant *Achromobacter xylosoxidans* using response surface methodology. Bioresour Technol.

[CR15] Hughes DE, Wimpenny JWT, Lloyd D, Norris R, Robbons DW (1971). The disintegration of micro-organisms. Methods in microbiology.

[CR16] Johnston GC, Ehrhardt CW, Lorincz A, Carter BLA (1979). Regulation of cell size in the yeast *Saccharomyces cerevisiae*. J Bacteriol.

[CR17] Kakimoto T, Shibatani T, Nishimura N, Chibata I (1971). Enzymatic production of L-citrulline by *Pseudomonas putida*. Appl Microbiol.

[CR18] Maran JP, Priya B (2015). Comparison of response surface methodology and artificial neural network approach towards efficient ultrasound-assisted biodiesel production from muskmelon oil. Ultrason Sonochem.

[CR19] Marchitan N, Cojocaru C, Mereuta A, Duca G, Cretescu I, Gonta M (2010). Modeling and optimization of tartaric acid reactive extraction from aqueous solutions: a comparison between response surface methodology and artificial neural network. Sep Purific Technol..

[CR20] Mei CY, Ti TB, Ibrahim MN, Ariff A, Chuan LT (2005). The disruption of *Saccharomyces cerevisiae* cells and release of glucose-6-phosphate dehydrogenase (G6PDH) in a horizontal Dyno bead mill operated in continuous recycling mode. Biotechnol Bioprocess Eng.

[CR21] Middelberg APJ, Desai MA (2000). Microbial cell disruption by high-pressure homogenization. Downstream processing of proteins: methods and protocols.

[CR22] Ni Y, Liu Y, Schwaneberg U, Zhu L, Li N, Li L, Sun Z (2011). Rapid evolution of arginine deiminase for improved anti-tumor activity. Appl Microbiol Biotechnol.

[CR23] Okungbowa FI, Ghosh AK, Chowdhury R, Chaudhuri P, Basu A, Pal K (2007). Mechanical lysis of *Candida* cells for crude proteinand enzymatic activity estimation: comparison of three methods. World J Med Sci..

[CR24] Panchaud N, Péli-Gulli MP, De Virgilio C (2013). Amino acid deprivation inhibits TORC1 through a GTPase-activating protein complex for the Rag family GTPase Gtr1. Sci Signal.

[CR25] Patil MD, Bhaumik J, Babykutty S, Banerjee UC, Fukumura D (2016). Arginine dependence of tumor cells: targeting a chink in cancer’s armor. Oncogene.

[CR26] Patil MD, Shinde KD, Patel G, Chisti Y, Banerjee UC (2016). Use of response surface method for maximizing the production of arginine deiminase by *Pseudomonas putida*. Biotechnol Rep.

[CR27] Pedregosa F, Varoquaux G, Gramfort A, Michel V, Thirion B, Grisel O, Blondel M, Prettenhofer P, Weiss R, Dubourg V, Vanderplas J, Passos A, Cournapeau D, Brucher M, Perrot M, Duchesnay E (2011). Scikit-learn: machine learning in Python. J Mach Learn Res.

[CR28] Ricci-Silva ME, Vitolo M, Abrahao-Neto J (2000). Protein and glucose 6-phosphate dehydrogenase releasing from baker’s yeast cells disrupted by a vertical bead mill. Process Biochem.

[CR29] Sarve A, Sonawane SS, Varma MN (2015). Ultrasound assisted biodiesel production from sesame (*Sesamumindicum* L.) oil using barium hydroxide as a heterogeneous catalyst: comparative assessment of prediction abilities between response surface methodology (RSM) and artificial neural network (ANN). Ultrason Sonochem.

[CR30] Shirai H, Blundell TL, Mizuguchi K (2001). A novel superfamily of enzymes that catalyse the modification of guanidine groups. Trends Biochem Sci.

[CR31] Singh RS (2013). A comparative study on cell disruption methods for release of aspartase from *E. coli* K-12. Ind J Exp Biol..

[CR32] Singh R, Banerjee A, Kaul P, Barse B, Banerjee UC (2005). Release of an enantioselective nitrilase from *Alcaligenes faecalis* MTCC 126: a comparative study. Bioprocess Biosyst Eng.

[CR33] Smola AJ, Scholkopf B (2004). A tutorial on support vector regression. Stat Comput..

[CR34] Tam YJ, Allaudin ZN, Lila MAM, Bahaman AR, Tan JS, Rezaei MA (2012). Enhanced cell disruption strategy in the release of recombinant hepatitis B surface antigen from *Pichia pastoris* using response surface methodology. BMC Biotechnol.

[CR35] Wangpaichitr M, Wu C, Bigford G, Theodoropoulos G, You M, Li Y, Verona-Santos J, Feun LG, Nguyen DM, Savaraj N (2014). Combination of arginine deprivation with TRAIL treatment as a targeted-therapy for mesothelioma. Anticancer Res.

[CR36] Witek-Krowiak A, Chojnacka K, Podstawczyk D, Dawiec A, Pokomeda K (2014). Application of response surface methodology and artificial neural network methods in modelling and optimization of biosorption process. Bioresour Technol.

